# Defining a taxonomy of Medicare-funded home-based clinical care using claims data

**DOI:** 10.1186/s12913-023-09081-8

**Published:** 2023-02-06

**Authors:** Claire K. Ankuda, Katherine A. Ornstein, Bruce Leff, Subashini Rajagopalan, Bruce Kinosian, Abraham A. Brody, Christine S. Ritchie

**Affiliations:** 1 Brookdale Department of Geriatrics and Palliative Medicine, Icahn School of Medicine at Mount Sinai, New York, NY USA; 2 Center for Transformative Geriatric Research, Johns Hopkins University School of Medicine, Baltimore, MD USA; 3 Department of Medicine, Hospital of the University of Pennsylvania, Philadelphia, PA USA; 4 Hartford Institute for Geriatric Nursing, NYU Rory Meyers College of Nursing, New York, NY USA; 5 NYU Grossman School of Medicine, New York, NY USA; 6 Mongan Institute Center for Aging and Serious Illness, Massachusetts General Hospital, 100 Cambridge Street, Suite 1600, Boston, MA USA

**Keywords:** Geriatrics, Home-based care, Medicare, Home health

## Abstract

**Background:**

As more Americans age in place, it is critical to understand care delivery in the home. However, data on the range of home-based services provided by Medicare is limited. We define a taxonomy of clinical care in the home funded through fee-for-service Medicare and methods to identify receipt of those services.

**Methods:**

We analyzed Fee-for-service (FFS) Medicare claims data from a nationally-representative cohort of older adults, the National Health and Aging Trends Study (NHATS), to identify home-based clinical care. We included 6,664 NHATS enrollees age ≥ 70 and living in the community, observed an average of 3 times each on claims-linked NHATS surveys. We examined provider and service type of home-based clinical care to identify a taxonomy of 5 types: home-based medical care (physician, physician assistant, or nurse practitioner visits), home-based podiatry, skilled home health care (SHHC), hospice, and other fee-for-service (FFS) home-based care. We further characterized home-based clinical care by detailed care setting and visit types.

**Results:**

From 2011–2016, 17.8%-20.8% of FFS Medicare beneficiaries age ≥ 70 received Medicare-funded home-based clinical care. SHHC was the most common service (12.8%-16.1%), followed by other FFS home-based care (5.5%-6.5%), home-based medical care (3.2%-3.9%), and hospice (2.6%-3.0%). Examination of the other-FFS home-based care revealed imaging/diagnostics and laboratory testing to be the most common service.

**Conclusions:**

We define a taxonomy of clinical care provided in the home, serving 1 in 5 FFS Medicare beneficiaries. This approach can be used to identify and address research and clinical care gaps in home-based clinical care delivery.

**Supplementary Information:**

The online version contains supplementary material available at 10.1186/s12913-023-09081-8.

## Introduction

Medical care for older adults with long-term care needs in the United States is increasingly moving into the home and community and away from hospitals and nursing facilities. The proportion of older adults who reside in nursing homes has fallen in the last decade, and more older adults with care needs are choosing to reside in the community, largely with family/friend assistance, or are moving into a variety of types of residential care facilities (e.g., assisted living) that provide varying degrees of assistance such as medication management [1–3]. Recognizing the preference of older adults and potential cost-savings of in-home care, Medicaid has substantially increased the proportion of long-term care spending in the home and community compared to institutional settings [4].

This shift to care in the home has resulted in growing provision of clinical care in home settings, from increasing home hospice, as more older adults die in their homes compared to hospitals, to growth in skilled home health use [5, 6]. This trend in care shifting to the home is only anticipated to increase given the potential savings of home-based clinical care [7] and the shift in attitudes towards facility-based long-term care that has resulted from the COVID-19 pandemic and avoidance of hospitals [8, 9]. This makes it important to understand the role of Medicare, the largest source of health insurance for older adults in the United States, in paying for care in the home.

Despite the growing significance of care in the home, to our knowledge the totality of fee-for-service Medicare-funded care delivered in the home has not previously been examined. Understanding and classifying the range of Medicare services at home is a necessary step for researchers and policy makers to comprehensively assess Medicare-funded care in the home, understand the drivers of how service delivery varies, and measure how service delivery patterns influence care outcomes. Within the context of fee-for-service Medicare, similar to within private health insurance plans, clinical services provided in the home address a broad spectrum of needs, from acute, to post-acute, to longitudinal [10]. To date, research on Medicare-funded home-based clinical care has been limited to specific service types, namely studies of skilled home health care [11], home-based medical care [12], and hospice [13]. Even published Medicare reports of services separately examine hospice and skilled home health and do not describe other Medicare-funded services delivered in the home or how they relate to each other [14]. These other home-based clinical services funded by Medicare, such as podiatry visits, home-based therapy provided outside the skilled home health benefit, and other home-based clinical services have not been captured in the literature on home-based clinical care. Given that there is strong regional variation in skilled home health [15], hospice [16], and home-based medical care [17], with particular growth in settings such as assisted living [18], it is important to measure the full array of home-based clinical care available to high-need populations and to identify gaps in care that need to be filled.

As clinical care increasingly moves into the home, we will require a system and consistent language for identifying and describing the landscape of home-based clinical care to further assess what care patterns improve outcomes for patients and their caregivers. We therefore aim to develop a taxonomy of Medicare-funded clinical care provided in the home using health care claims linked to a nationally representative survey of aging. We offer a classification schema for these services and assess the size and scope of the population that they reach. We will outline this approach and provide guidance for other researchers looking to expand their investigation in home-based clinical care.

## Methods

### Data and cohort

We used fee-for-service Medicare claims years 2011 to 2017 linked to an annual nationally-representative cohort study, the National Health and Aging Trends Study (NHATS), 2011–2016. This allowed us to identify home-based clinical care provided by Medicare and provide national estimates on rates and trends, given that NHATS allows for estimating nationally-representative estimates across years through survey weights and design parameters [19]. NHATS itself is a critical resource for aging research, particularly for research on the care, context, and outcomes among older adults with functional disability [20, 21]. In addition, the structure of the claims-linked NHATS survey is similar to other important population-based studies of aging, such as the Medicare Current Beneficiary Survey and the Health and Retirement Study, allowing for future application of the approach to these cohorts [22–24].

We limited the NHATS cohort to adults age 70 and older, residing in the community (i.e., not in a long-term nursing facility) since our goal was to characterize clinical care received in a home setting. While Medicare eligibility for older adults starts at age 65, we limited to age 70 and older to create a nationally representative sample across survey waves of NHATS given that NHATS has refreshed its cohort every 5 years and so older adults between the age of 65 and 69 are not captured in every survey year [19]. We further restricted the sample to only include those with at least 1 month of fee-for-service Medicare per calendar year in order to identify home-based clinical care via claims.

### Defining a taxonomy of home-based clinical care provided by Medicare

Within each Medicare claims file where we might identify clinical care in the home (the outpatient, carrier, hospice, and home health files), we first limited to observations of home-based services. We then proceeded to characterize each service in the home in terms of the type of clinical visit provided, thus developing a taxonomy of home-based clinical care.

We first classified three categories of home-based care that have been independently described in the literature: home-based medical care (i.e. house calls, physician home visits), hospice, and home health. We relied on published literature to define home-based medical care [12, 17] and hospice visits [25], as well as Medicare claims processing guides which specified how to identify care of these types delivered in the patient’s home. Where possible, we identified how services could be differentiated with even more specificity in terms of where they are provided, such as in a private home vs. domiciliary (generally an assisted living or custodial care facility). We also examined differences in clinician specialty which allowed us to better differentiate types of home-based clinical care, e.g., identification of home-based podiatry visits. Home-based podiatry was separately examined given that for this common home-based service, podiatrists use the same claims codes as do home-based medical care providers, but have distinct clinician specialty codes.

### Examination of other FFS Medicare clinical visits in the home

**Fig. 1:**
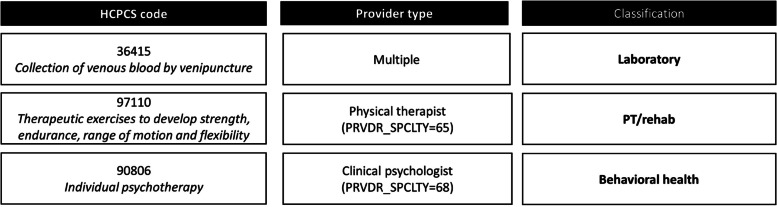
Approach to classifying other FFS claims for home-based care: example HCPCS and providers Legend: FFS = fee-for-service, or Traditional Medicare; HCPCS = healthcare common procedure coding system

We then sought to better understand and classify the billing codes comprising the “other FFS home” category: those in the carrier file and with a location of home but without Healthcare Common Procedure Coding System (HCPCS) codes defining home-based medical care or home-based podiatry. These visits occurred in the home but had not previously been defined or examined. To characterize these, we examined the most frequent HCPCS codes for these services until we had captured 80% of claims. We used an iterative process to categorize these claims, as many were similar: for example, we categorized HCPCS for gait training to be physical therapy related; HCPCS for thyroid levels and complete blood counts to be laboratory related. These claims were categorized by two independent researchers, one with public health training (S.R.) and one a physician-investigator (C.A.). When there was disagreement or uncertainty about the categorization of a HCPCS it was reviewed by a home-based care epidemiologist (K.O.) and geriatrician investigator with research and clinical experience in home-based clinical care (C.R.). Finally, when relevant, the provider type for the clinician billing the claim was cross-referenced against the HCPCS categorization, for example HCPCS categorized as podiatry-related were assessed to see if the clinician was a podiatrist (see Fig. 1 for additional examples). We also examined the provider type for each HCPCS using not only the NHATS claims data but the publicly-available Medicare Public Use Files [26]. For complete details of all HCPCS and assigned categories see supplementary appendix.

### Analysis

We estimated the proportion of the cohort receiving each type of home-based care in the 12 months after NHATS interview, which allowed us to estimate a national estimate for the proportion of adults age ≥ 70 receiving home-based care. In order to understand how including each service expanded the scope of home-based care, we then compared the overlap of home-based medical care, skilled home health, and other FFS home-based care. Finally, we examined rates of home-based care by time, testing to see if any service category increased from 2011 to 2017. All analyses other than that of temporal trends accounted for clustering of multiple observations per person. All analyses additionally accounted for survey design and weighting for differential response and oversampling in order to generate nationally-representative estimates of utilization [19].

## Results

**Fig. 2:**
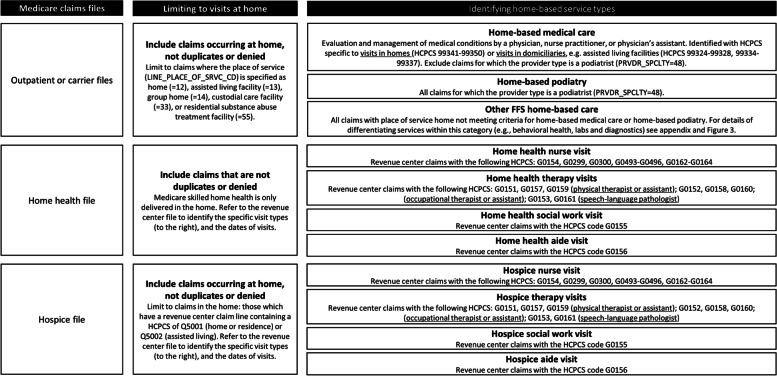
Identification of Medicare-funded home-based clinical care Legend: FFS = fee-for-service, or Traditional Medicare; HCPCS = healthcare common procedure coding system

**Fig. 3:**
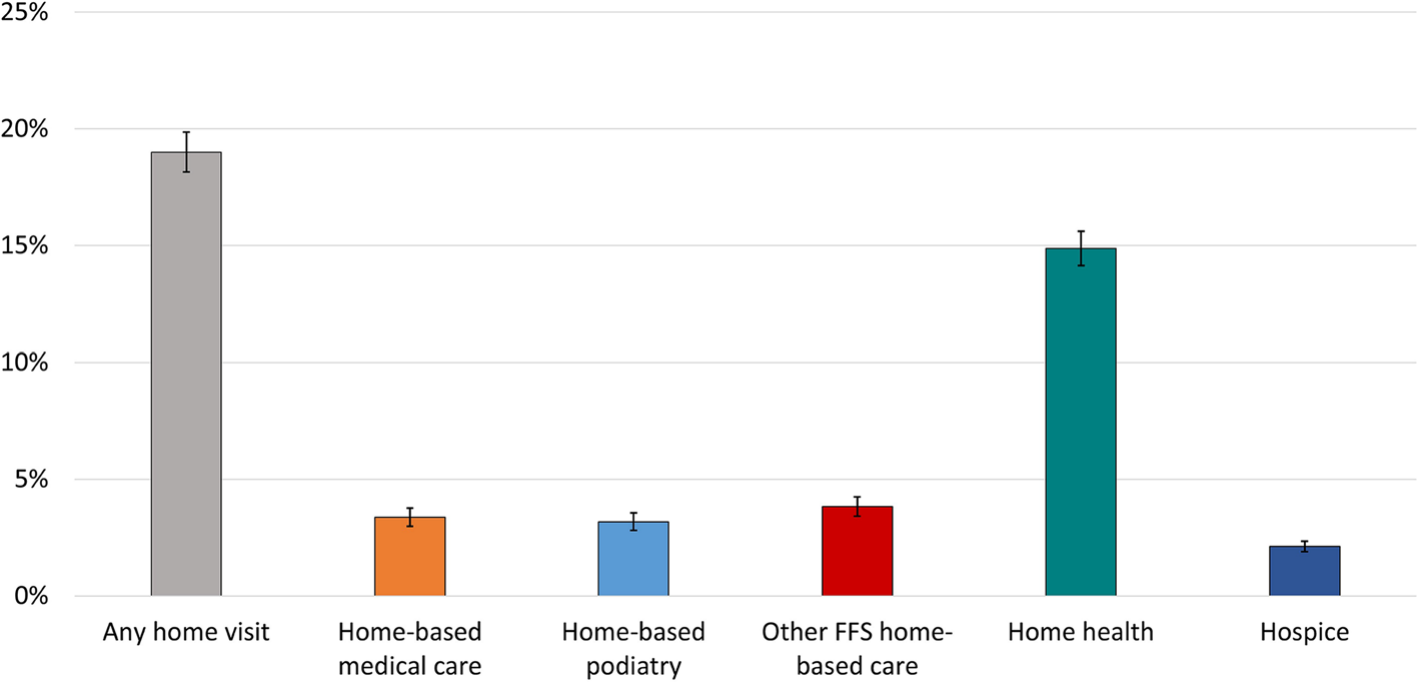
Medicare provision of care in the home. Legend: Data source: National Health and Aging Trends Study, 2011–2017

Using Medicare claims, we describe a taxonomy of all home-based clinical care, identified in the outpatient or carrier, hospice, and home health files (Fig. 2). In order to assess the patterns of these services in a representative cohort, we then identified 6,664 NHATS respondents from 2011–2016 with at least one month of FFS Medicare claims after their NHATS survey, each observed an average of 3 times on annual NHATS surveys. As demonstrated in Fig. 3, 19.0% of older adults received any type of Medicare-provided clinical care at home the year after NHATS survey. Skilled home health care was the most common services provided in the home (received by 14.9%), followed by other FFS home-based care (received by 3.8%), home-based medical care (received by 3.4%), home-based podiatry (received by 3.2%) and hospice (received by 2.1%).

**Fig. 4:**
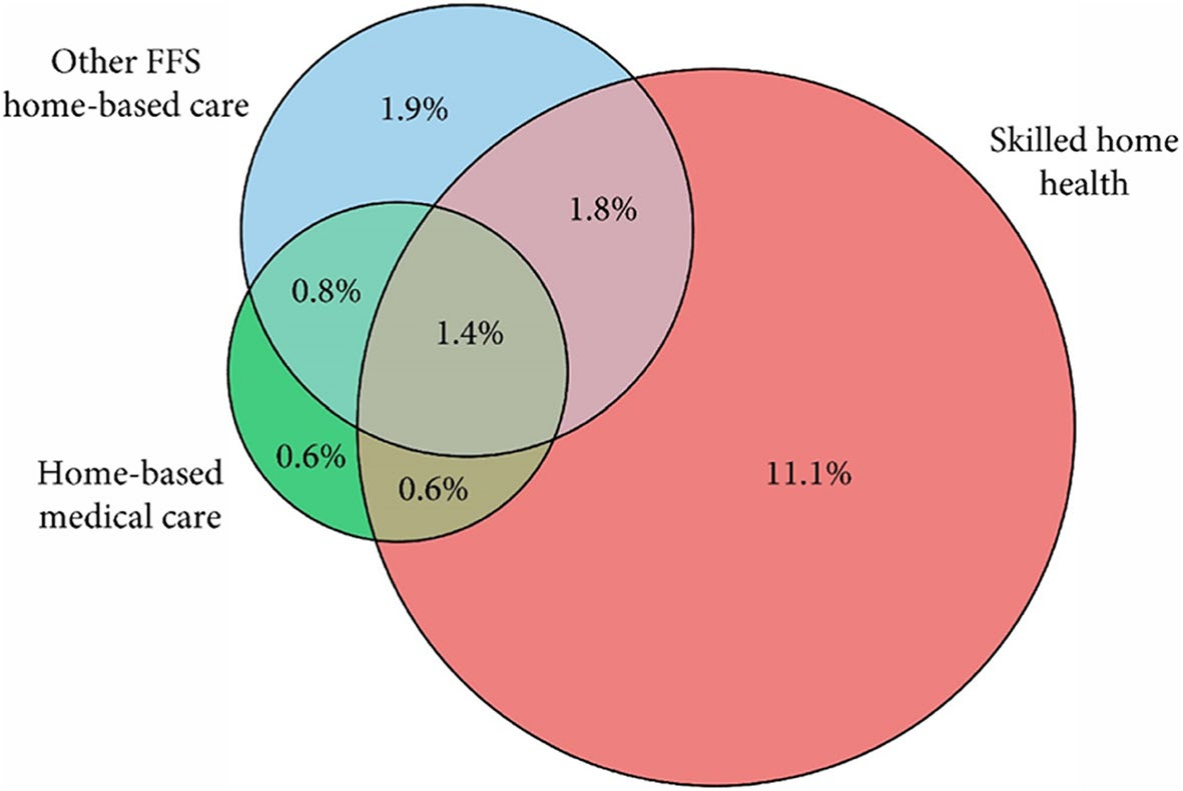
Overlap between the populations receiving Medicare skilled home health care, Medicare home-based medical care, and other FFS Medicare home-based care. Legend: Data source: National Health and Aging Trends Study, 2011–2017. Other FFS home-based care is any visit in the home not meeting criteria for home-based medical care. For the purpose of this Venn diagram, home-based podiatry is included in “Other FFS home-based care”

Figure 4 demonstrates the overlap in populations receiving three categories of services: skilled home health, home-based medical care, and other FFS home-based clinical care. As demonstrated, the majority (84.9%) of those receiving home-based medical care receive additional home-based care in the form of skilled home health (15.8%), other FFS home-based care (22.3%) or both (46.8%). However, among those with skilled home health, only 27.1% additionally received home-based medical care and/or other FFS home-based care. In addition, while skilled home health was the most common service, 18.1% who received clinical care at home (either home-based medical care or other FFS home-based care) did not receive skilled home health.

When we further examined the other services that FFS Medicare provides in the home (Fig. 1), we identify a range of services. The two most common, imaging/diagnostics and laboratory tests in the home, were provided to 1.5% and 1.3% of respondents respectively. Next common were services that supplemented Medicare skilled home health such as medication management/care coordination and certification/recertification and care plan oversight, as well as therapy and rehab (most commonly provided by a physical or occupational therapist) and behavioral health. Notably, we identified these physical and occupational therapy visits in this category as a stand-alone service funded through Part B, as well as within skilled home health and hospice as part of those benefits, illustrating that this service exists across multiple claims files and Medicare programs.

Among each claim category of home-based care, we were able to identify multiple sub-categories of care, which are described in Fig. 1 and detailed in the Supplementary Appendix. These include the provider type; the location of the patient in terms of home or a domiciliary, and then in more detail in the case of skilled home health and hospice; and the specific visit types in the other FFS category, skilled home health, and hospice.

## Discussion

Using Medicare claims data, we defined a taxonomy for the full spectrum of clinical care provided in the home, including both previously defined services (e.g. skilled home health, home-based medical care) and home-based care not consistently or widely described in the literature (e.g. podiatry home visits, non-home health PT, home-based behavioral health, diagnostic and imaging services). This classification and full description of Medicare services may be used by other researchers as a methodological framework to better understand and evaluate trends and impact of home-based clinical care. Applying this approach to the NHATS survey linked to Medicare claims, we identified that 19% of the population of older adults age ≥ 70 received care in the home between 2012 and 2017.

These methods can be used as a standard to identify a range of home-based care through Medicare that a focus on any one specific program might miss. We also found that a “full-spectrum” approach to capturing care in the home was important as the same service types could be delivered through multiple mechanisms. For example, home health aides may be provided via skilled home health care or hospice benefits. Physical therapy may be provided as part of a skilled home health episode or as a stand-alone service under Part B. Podiatry care may be identified using the codes defining home-based medical care with a podiatrist as the provider in the carrier file, or through identifying care with a place of service as home and a podiatry specialist in the carrier file.

In examining the overlap in populations receiving each type of home-based care, we identified heterogeneity of services received, with many respondents receiving only skilled home health, others receiving various combinations of other FFS home-based care, home-based medical care, and hospice. It is possible that some of this variation is due to differences in clinical context: for example, a person undergoing a hospitalization for a hip replacement might require skilled home health for wound care and physical therapy, but little other services after recovery. However, particular examination of which home-based care services are provided to which higher risk populations such as those with dementia, persistent functional disability, and serious illness will be important to understand how home-based care is delivered or tailored to specific patient need. In addition, it will be important to assess how these services do or do not coordinate with Medicaid-funded long-term care, particularly for the growing number of adults in managed Medicaid plans which theoretically have a greater investment in coordinating all services in the home. While examining Medicaid-funded home-based services is outside the scope of this manuscript, it will be important to understand how Medicare and Medicaid services interact, which may vary considerably by State Medicaid program. The heterogeneity of Medicare-funded clinical services also warrants further examination of non-clinical drivers of variation, including race, socioeconomics, and regional factors, and the association of different patterns of home-based care use to outcomes such as unmet health needs, hospitalization, and institutionalization in nursing facilities.

This work demonstrates an approach to using FFS Medicare claims to identify a range of clinical services in the home funded by Medicare but does not examine potential changes in rates of services over time or since the beginning of the COVID-19 pandemic. However, our taxonomy may be applied to better assess temporal trends in service delivery. As noted above, provision of services via Medicaid is not included in this taxonomy. We do not include the provision of durable medical equipment in the home, as we focused more on clinical visits delivered in the home. This work is the first to provide substantial granularity on the spectrum of home-based clinical care services provided through Medicare fee-for-service; to date the only other work exploring home-based clinical care was within Medicare Advantage and commercial plans and not in the Medicare fee-for-service context [10]. It will be increasingly important to contrast services provided by MA, given that Medicare Advantage has expanded flexibility to provide non-traditional services in the home, but also increased incentive to reduce costs of care [27, 28]. We do not capture services provided through additional insurance programs or supports such as commercial insurers, Veteran’s Health Administration benefits or long-term care insurance. Further work to map this proposed taxonomy to data from other insurers will advance the study of clinical care in the home. While we have nuanced information on the setting of individuals at the time of NHATS survey, some of the cohort may have died or moved into nursing facilities before the end of 12 months thereby underestimating receipt of home-based care. Our study does not capture the experience of older adults age 65–69 given NHATS’ survey design. It is critical that further work capture the full breadth of care in the home as well as the costs of different types of care provided. Finally, as these data were collected prior to 2020, we do not simultaneously assess telemedicine as a type of care provided to individuals in the home that expanded during COVID-19, which is likely an important aspect of care for this population [29], or the new Hospital at Home Medicare waiver [30]. These and other home-based clinical services must be considered in future work.

We offer technical guidance to measure the range of home-based clinical care funded by Medicare. This is only one piece of the services and supports that older adults, especially those with complex health and caregiving needs, require to age in place. However, the impact of the evolving COVID-19 pandemic and the incentives and opportunity provided by the growth in Medicare Advantage will likely continue shifting care from hospital and nursing facilities to the home. If we are to better serve the population of older adults at home, we must make strides in measuring the full spectrum of clinical care delivered in the home and ultimately the quality of care delivery.

## Supplementary Information

**Additional file 1. Supplementary Appendix:** Complete list of the most common HCPCS found on other FFS home-based clinical care and their categorization.

## Data Availability

The data that support the findings of the study are available from NHATS but restrictions apply to some elements of these data, including Medicare claims, which were used under a data use agreement for the current study, and so are not publicly available. Data are available from www.NHATS.org with permission and approvals.
