# Supplementary material for: Defining a taxonomy of Medicare-funded home-based clinical care using claims data
Source: BMC Health Serv Res. 2023 Feb 6;23:120. doi: 10.1186/s12913-023-09081-8 (PMC9900204; doi:10.1186/s12913-023-09081-8)
Supplement: Supplementary file 1 — Additional file 1. Supplementary Appendix: Complete list of the most common HCPCS found on other FFS home-based clinical care and their categorization. [file 12913_2023_9081_MOESM1_ESM.docx]

Supplementary Appendix:

Complete list of the most common HCPCS found on other FFS home-based clinical care and their categorization

| **Category** | **Description** | **HCPCS** | **Frequency** |
| --- | --- | --- | --- |
| Podiatry* | Surgical Procedures on the Nails | 11721 | 1249 |
| Laboratory | Collection of venous blood by venipuncture | 36415 | 937 |
| PT/rehab | Therapeutic exercises to develop strength, endurance, range of motion and flexibility. | 97110 | 820 |
| Laboratory | Travel allowance one way in connection with medically necessary laboratory specimen collection drawn from home bound or nursing home bound patient; prorated trip charge | P9604 | 749 |
| PT/rehab | use of dynamic activities to improve functional performance. Examples of such activities include lifting, pushing, pulling, reaching, throwing, etc. | 97530 | 729 |
| PT/rehab | Therapeutic Procedure | 97112 | 437 |
| Imaging/diagnostics | Set-up portable x-ray equipment | Q0092 | 346 |
| Laboratory | Travel allowance one way in connection with medically necessary laboratory specimen collection drawn from home bound or nursing home bound patient; prorated miles actually travelled | P9603 | 320 |
| Imaging/diagnostics | Transportation of portable x-ray equipment and personnel to home or nursing home, per trip to facility or location, one patient seen | R0070 | 288 |
| PT/rehab | Gait Training | 97116 | 283 |
| Podiatry* | Paring or Cutting Procedures on the Skin | 11056 | 268 |
| Podiatry* | Surgical Procedures on the Nails | 11720 | 267 |
| PT/rehab | Manual therapy techniques, such as mobilization and manipulation, manual lymphatic drainage, and manual traction. | 97140 | 239 |
| Podiatry* | Paring or Cutting Procedures on the Skin | 11055 | 199 |
| Behavioral health | Psychotherapy, 45 minutes with patient | 90834 | 193 |
| Medication management/care coordination | Eligible clinician attests to documenting in the medical record they obtained, updated, or reviewed the patient's current medications | G8427 | 185 |
| Certification/Recertification and Care Plan Oversight | Physician or allowed practitioner certification for medicare-covered home health services under a home health plan of care (patient not present), including contacts with home health agency and review of reports of patient status required by physicians and allowed practitioners to affirm the initial implementation of the plan of care | G0180 | 165 |
| Certification/Recertification and Care Plan Oversight | Physician or allowed practitioner supervision of a patient receiving medicare-covered services provided by a participating home health agency (patient not present) requiring complex and multidisciplinary care modalities involving regular physician or allowed practitioner development and/or revision of care plans | G0181 | 159 |
| Behavioral health | Individual Psychotherapy | 90806 | 152 |
| Imaging/diagnostics | Radiologic examination, chest, single view, frontal | 71010 | 142 |
| Certification/Recertification and Care Plan Oversight | Physician or allowed practitioner re-certification for medicare-covered home health services under a home health plan of care (patient not present), including contacts with home health agency and review of reports of patient status required by physicians and allowed practitioners to affirm the initial implementation of the plan of care | G0179 | 138 |
| Podiatry* | Surgical Procedures on the Nails | 11719 | 137 |
| PT/rehab | Self-Care/Home Management Training | 97535 | 126 |
| Medication management/care coordination | Non-complex CCM is a 20-minute timed service provided by clinical staff to coordinate care across providers and support patient accountability. | 99490 | 126 |
| Imaging/diagnostics | radiologic examination, chest, two views, frontal and lateral | 71020 | 103 |
| Podiatry* | Trimming of dystrophic nails, any number | G0127 | 95 |
| PT/rehab | Mobility: walking & moving around functional limitation, projected goal status, at therapy episode outset, at reporting intervals, and at discharge or to end reporting | G8979 | 88 |
| Podiatry* | Wound debridement codes (not associated with fractures) | 11042 | 87 |
| PT/rehab | Mobility: walking & moving around functional limitation, current status, at therapy episode outset and at reporting intervals | G8978 | 75 |
| Behavioral health | 60 minute individual psychotherapy | 90837 | 71 |
| Imaging/diagnostics | Implantable, Insertable, and Wearable Cardiac Device Evaluations | 93296 | 71 |
| Imaging/diagnostics | Echocardiography, transthoracic, real-time with image documentation (2D) | 93306 | 70 |
| Laboratory | Glucose, blood by glucose monitoring device(s) cleared by the FDA specifically for home use | 82962 | 56 |
| Imaging/diagnostics | Implantable, Insertable, and Wearable Cardiac Device Evaluations. | 93294 | 55 |
| Behavioral health | Individual psychotherapy, insight oriented, behavior modifying and/or supportive, 30 minutes with the patient and/or family member (time range 16-37 minutes), when performed with an evaluation and management service. | 90833 | 52 |
| Imaging/diagnostics | Complete bilateral noninvasive physiologic studies of upper or lower extremity arteries, 3 or more levels. | 93923 | 49 |
| Behavioral health | Psychotherapy, 30 minutes with patient | 90832 | 48 |
| Podiatry* | Paring or cutting of benign hyperkeratotic lesion (eg, corn or callus) | 11057 | 46 |
| Imaging/diagnostics | Lower extremity arteries or arterial bypass grafts; complete bilateral study | 93925 | 44 |
| Behavioral health | Individual Psychotherapy | 90808 | 43 |
| PT/rehab | Physical Therapy (PT) Evaluation | 97001 | 42 |
| Laboratory | When physicians use a prothrombin time test (reported with CPT code 85610) to monitor patients on anticoagulant drugs | 85610 | 41 |
| Imaging/diagnostics | Duplex scan of extremity veins including responses to compression and other maneuvers; complete bilateral study | 93970 | 41 |
| Behavioral health | Interactive Complexity (90785) is an add-on code specific for psychiatric services and refers to communication difficulties during the psychiatric procedure | 90785 | 40 |
| Imaging/diagnostics | Noninvasive physiologic studies of extremity veins, complete bilateral study (eg, doppler waveform analysis with responses to compression and other maneuvers, phleborheography, impedance plethysmography) | 93965 | 39 |
| Podiatry* | Avulsion of the nail plate whether partial or complete uses the CPT code CPT 11730 | 11730 | 38 |
| Medication management/care coordination | Home visit for the evaluation and management of a new patient, which requires these 3 key components: A detailed history; A detailed examination; and. Medical decision of moderate complexity. | 99343 | 36 |
| Imaging/diagnostics | Duplex scan of extracranial arteries; complete bilateral study | 93880 | 34 |
| PT/rehab | Ultrasound/Phonophoresis | 97035 | 34 |
| Behavioral health | Psychiatric Therapeutic Procedures | 90804 | 32 |
| Imaging/diagnostics | Transportation of portable x-ray equipment and personnel to home or nursing home, per trip to facility or location, more than one patient seen | R0075 | 32 |
| Laboratory | Collection of venous blood by venipuncture or urine sample by catheterization from an individual in a skilled nursing facility (snf) or by a laboratory on behalf of a home health agency (hha) | G0471 | 31 |
| Imaging/diagnostics | Electrocardiogram, routine ECG with at least 12 leads. | 93000 | 30 |
| Medication management/care coordination | Administration of influenza virus vaccine | G0008 | 30 |
| Medication management/care coordination | Physician review, interpretation, and patient management of home inr testing for patient with either mechanical heart valve(s), chronic atrial fibrillation, or venous thromboembolism who meets medicare coverage criteria; testing not occurring more frequently than once a week; billing units of service include 4 tests | G0250 | 30 |
| Imaging/diagnostics | Diagnostic Radiology (Diagnostic Imaging) Procedures of the Spine and Pelvis | 72100 | 28 |
| PT/rehab | Self care functional limitation, projected goal status, at therapy episode outset, at reporting intervals, and at discharge or to end reporting or just “Self care goal status” for short, used in Medical care | G8988 | 28 |
| Medication management/care coordination | Patient History | 1036F | 27 |
| PT/rehab | Changing & maintaining body position functional limitation, projected goal status, at therapy episode outset, at reporting intervals, and at discharge or to end reporting | G8982 | 25 |
| Laboratory | Blood count; complete (CBC), automated (Hgb, Hct, RBC, WBC and platelet count) | 85025 | 24 |
| Imaging/diagnostics | Implantable, Insertable, and Wearable Cardiac Device Evaluations. | 93295 | 22 |
| PT/rehab | Self care functional limitation, current status, at therapy episode outset and at reporting intervals | G8987 | 22 |
| Imaging/diagnostics | Duplex scan of extremity veins including responses to compression and other maneuvers | 93971 | 21 |
| PT/rehab | Occupational therapy evaluation | 97003 | 21 |
| PT/rehab | Wheelchair management (eg, assessment, fitting, training), each 15 minutes | 97542 | 21 |
| Medication management/care coordination | Physician Quality Reporting System (PQRS) code used to indicate the patient encounter was documented using a certified electronic health record (EHR) | G8447 | 21 |
| PT/rehab | Functional outcome assessment documented as positive using a standardized tool and a care plan based on identified deficiencies on the date of functional outcome assessment | G8539 | 21 |
| Medication management/care coordination | Pain assessment using a standardized tool is documented as negative, no follow-up plan required | G8731 | 21 |
| Medication management/care coordination | Annual wellness visit, includes a personalized prevention plan of service (pps), subsequent visit | G0439 | 20 |
| Imaging/diagnostics | Radiologic examination, hip, unilateral | 73510 | 19 |
| Imaging/diagnostics | Diagnostic Radiology (Diagnostic Imaging) Procedures of the Lower Extremities | 73620 | 19 |
| Laboratory | Thyroid Testing | 84443 | 19 |
| Podiatry* | wet-to-dry dressings, application of medications with enzymes to dissolve dead tissue, whirlpool baths, minor removal of loose fragments with scissors, scraping away tissue with sharp instruments, debridement with pulse lavage, high-pressure irrigation, incision, and drainage. | 97597 | 19 |
| Imaging/diagnostics | Lower extremity neurological exam performed and documented | G8404 | 19 |
| PT/rehab | Changing & maintaining body position functional limitation, current status, at therapy episode outset and at reporting intervals | G8981 | 19 |
| Imaging/diagnostics | Diagnostic Radiology (Diagnostic Imaging) Procedures of the Lower Extremities. | 73610 | 18 |
| PT/rehab | Chiropractic manipulative treatment (CMT); spinal, 1-2 regions. | 98940 | 18 |
| Imaging/diagnostics | Miscellaneous supply or accessory for use with an implanted ventricular assist device | Q0508 | 18 |
| PT/rehab | Patient screened for future fall risk; documentation of no falls in the past year or only one fall without injury in the past year . | 1101F | 17 |
| Medication management/care coordination | Bmi is documented within normal parameters and no follow-up plan is required | G8420 | 17 |
| Imaging/diagnostics | Diagnostic Radiology (Diagnostic Imaging) Procedures of the Lower Extremities | 73560 | 16 |
| PT/rehab | Physical performance test or measurement (eg, musculoskeletal, functional capacity), with written report, each 15 minutes. | 97750 | 16 |
| Imaging/diagnostics | Home sleep test (hst) with type iii portable monitor, unattended; minimum of 4 channels: 2 respiratory movement/airflow, 1 ecg/heart rate and 1 oxygen saturation | G0399 | 16 |
| Medication management/care coordination | Current list of medications not documented as obtained, updated, or reviewed by the eligible clinician, reason not given | G8428 | 16 |
| PT/rehab | Mobility: walking & moving around functional limitation, discharge status, at discharge from therapy or to end reporting | G8980 | 16 |
| Laboratory | Comprehensive metabolic panel | 80053 | 15 |
| *These claims were additionally classified as being podiatry-related services through their associated provider type (PRVDR_SPCLTY=48) | | | |
